# DNA Methylation Markers for Pan-Cancer Prediction by Deep Learning

**DOI:** 10.3390/genes10100778

**Published:** 2019-10-04

**Authors:** Biao Liu, Yulu Liu, Xingxin Pan, Mengyao Li, Shuang Yang, Shuai Cheng Li

**Affiliations:** 1 BGI Education Center, University of Chinese Academy of Sciences, Shenzhen 518083, China; biaoliu2019@gmail.com (B.L.); liuyulu@genomics.cn (Y.L.);; 2 Research and Development Department, Shenzhen Byoryn Technology Co.,Ltd, Shenzhen 518000, China; limengyao007@gmail.com; 3 Department of Computer Science, City University of Hong Kong, Kowloon 999077, Hong Kong, China

**Keywords:** biomarker, methylation, pan-cancer, deep learning, CpG, promoter

## Abstract

For cancer diagnosis, many DNA methylation markers have been identified. However, few studies have tried to identify DNA methylation markers to diagnose diverse cancer types simultaneously, i.e., pan-cancers. In this study, we tried to identify DNA methylation markers to differentiate cancer samples from the respective normal samples in pan-cancers. We collected whole genome methylation data of 27 cancer types containing 10,140 cancer samples and 3386 normal samples, and divided all samples into five data sets, including one training data set, one validation data set and three test data sets. We applied machine learning to identify DNA methylation markers, and specifically, we constructed diagnostic prediction models by deep learning. We identified two categories of markers: 12 CpG markers and 13 promoter markers. Three of 12 CpG markers and four of 13 promoter markers locate at cancer-related genes. With the CpG markers, our model achieved an average sensitivity and specificity on test data sets as 92.8% and 90.1%, respectively. For promoter markers, the average sensitivity and specificity on test data sets were 89.8% and 81.1%, respectively. Furthermore, in cell-free DNA methylation data of 163 prostate cancer samples, the CpG markers achieved the sensitivity as 100%, and the promoter markers achieved 92%. For both marker types, the specificity of normal whole blood was 100%. To conclude, we identified methylation markers to diagnose pan-cancers, which might be applied to liquid biopsy of cancers.

## 1. Introduction

DNA methylation, as an important epigenetic modification, is associated with gene silencing, and the primary methylated sequence in vertebrates is CpG [1,2]. CpG methylations located at promoter silence the promoter activity, thus, they are negative correlated with the gene expression [3,4]. Furthermore, promoter methylations play major roles in cancers by suppressing transcription of some vital genes, such as tumor suppressor genes [5,6].

Since DNA methylation plays an important role in cancers, many studies have utilized DNA methylated sequences as biomarkers for cancer detections, including CpG markers and promoter markers. Specifically, irregular methylations in promoters of cancer-related genes could serve as biomarkers for early cancer diagnosis and prognosis [7]. For example, adenomatous polyposis coli (APC) promoter methylation could be a biomarker for early diagnosis of prostate cancer [8], and O6-methylguanine-DNA-methyltransferase (MGMT) promoter methylation might be a predictive biomarker for cancer prognosis [9]. For CpG markers, ten diagnosis markers and eight prognosis markers in circulating tumor DNA of hepatocellular carcinoma have been screened [10].

Although quite a few DNA methylation biomarkers have been identified, and some of them have even been commercialized [11], one of the common limits is that these markers can only apply to one or few cancer types. Studying the similarities and differences among diverse cancer types is known as pan-cancer analysis, which has revealed that some different cancer types could have similar methylation patterns, and biomarkers that cross boundaries among diverse cancer types are expected to be identified [12,13]. Although some pan-cancer differentially methylated CpG sites have been identified [14,15], effective and practical pan-cancer methylation biomarkers remain to be identified. In this study, we focused on identifying DNA methylation biomarkers, including CpG markers and promoter markers, for diagnosing pan-cancers. We collected the whole genome methylation data of 27 cancer types containing 10,140 cancer samples and 3386 normal samples from TCGA (The Cancer Genome Atlas) [12] and GEO (Gene Expression Omnibus) [16]. Then, we used machine learning to analyze and identify cancer-special CpG markers and promoter markers. Specifically, we constructed diagnostic prediction models by deep learning. Finally, we identified 12 CpG markers and 13 promoter markers, which can be used to predict pan-cancer precisely.

## 2. Materials and Methods 

### 2.1. Datasets

We totally collected whole genome methylation data of 10,140 cancer samples and 3386 normal samples from TCGA and GEO. Specifically, methylation data of 4840 cancer samples and 1742 matched normal samples (matched normal sample: healthy tissue adjacent to tumor from the same patient) were divided randomly into a training data set (named as Training data set), a validation data set (named as Validation data set), and a test data set (named as Test data set 1) (Table S1). To make markers more adaptive in virtual normal samples, we added 727 cancer samples and 836 normal samples (most of them were virtual normal samples) into the Training data set (virtual normal sample: healthy tissue from healthy, unrelated individuals). Therefore, the Training data set contained 4827 cancer samples and 2716 normal samples (Tables S2 and S3). Both Validation data set and Test data set 1 contained 370 cancer samples and 201 matched normal samples from eight cancer types (Tables S1 and S2); The other two test data sets are named as Test data set 2 and Test data set 3. Test data set 2 contained 3041 cancer samples and 268 matched normal samples from 15 cancer types (Table S4); Test data set 3 contained 1532 cancer samples and 540 virtual normal samples from five cancer types (Table S5). The methylation data of each sample came from Illumina’s Infinium HumanMethylation450 BeadChip, which contains more than 450,000 methylation sites. The details of all samples are summarized in Tables S2–S6. We calculated the average methylation beta value of all CpG sites located in the promoter of the same gene as methylation beta value of the promoter. Specifically, upstream 1500 bp of TSS (Transcription start site) to downstream 500 bp of TSS are defined as a promoter [17,18]. We removed CpG sites and promoters where at least one sample had missed value to guarantee more strict data sets. Finally, 139,422 of 485,000 CpG sites and 15,316 of 24,062 promoters were left for the following analysis. Therefore, all samples had 139,422 common CpG sites and 15,316 common promoters. Data containing CpG sites and data containing promoters were analyzed parallelly in the following steps.

### 2.2. Identifying Markers

For mining markers, first we used the ‘moderated t-statistics’ method [19] to conduct the prescreening procedure to get the methylation sites with the most differential methylation expression. This method utilized Empirical Bayes for shrinking the variance and Benjamini–Hochberg procedure [20] to adjust *p* values. We sorted all candidate markers by the adjusted *p* values from low to high (lower adjusted *p* value means that the differential rates of methylation between cancer samples and normal samples are larger), and we took the top 2000 markers as the next candidate markers. Next, we used two strategies to obtain fewer markers. One machine learning strategy is LASSO (least absolute shrinkage and selection operator) [21] under a binomial distribution. We randomly subsampled 75 percent of the samples every time and conducted LASSO procedure to identify markers with the biggest methylation beta value difference. After 1000 times sampling, we selected the markers that were chosen by LASSO at least 750 times. In this process, we did not choose the minimum lambda but chose the “1-se” lambda which is one standard error larger than the minimum to make the model simpler. Besides, the minimization goal we chose was ‘auc’ to make our model more robust. 10-fold cross-validation was applied each time. Another machine learning strategy is a random forest. The tree number to use for the first forest was 5000 and for all additional forests was 2000. The algorithm we applied used OOB (Out-of-bag) error as minimization criterion, and removed those least important variables from the random forest [22]. At each iteration, we set the dropping fraction of variables at 0.3. Four main R packages (‘limma’, ‘glmnet’, ‘doParallel’, and ‘varSelRF’) were implemented in R version 3.5.0 to conduct these three machine learning strategies.

### 2.3. Constructing Diagnostic Prediction Models

To construct diagnostic prediction models, we constructed two multi-layer feedforward neural networks, both of which contained one input layer, multiple hidden layers and one output layer. The source code we used for prediction is publicly available at https://github.com/BiaoLiu2017/Cancer-methylation. The input layer was namely the input data matrix (data matrices only containing marker sites), and the output layer had just one neural unit, whose activation function was sigmoid activation function while the activation function of hidden units was ReLU. For each hidden layer, the number of hidden units was the same. The cost function of the neural network was standard logistic regression cost function. The optimization algorithm we deployed in the network was Adam optimization algorithm, and the exponential decay rate for the first moment estimates was 0.9, while the second was 0.999. The learning rate decay strategy was an exponential decay, which means the learning rate would multiple a decay rate after specified epochs. To prevent overfitting, we carried out a batch normalization after activation function of every hidden layer. Another strategy to prevent overfitting is early stopping, and we chose a befitting training point to stop to make the model more suitable for the Validation data set. We conducted a random search [23] for hyper-parameter optimization. Table S7 shows the hyper-parameters we tuned in the process of training the neural network. In other words, we adopted the strategy that randomly initializes these hyper-parameters in the range as Table S7. We parallelly trained 1000 neural networks, and finally chose the hyper-parameters combination that had the best performance for the Validation data set. The final hyper-parameters combination is the best scheme as Table S7 shows. We deployed the best hyper-parameters into the final deep learning models and trained them by feeding the Training data set. We used the Validation data set to justify whether the model was overfitting. After we trained two neural network models whose performance were good enough in the Validation data set, we tested our diagnostic prediction models in Test data set 1. Furthermore, to evaluate the performance of our model unbiasedly, we tested our prediction models in the other two test data sets: Test data set 2 and Test data set 3. What needs to be emphasized is that all three test data sets were tested just once. The reason we divided samples in this way was to evaluate whether our model could predict untrained cancers. Before being fed into deep learning models, all data were subjected to standardization to fit a standardized normal distribution; namely the average was 0, and the standard deviation was 1. The deep neural network models that we deployed were based on the deep learning framework Tensorflow-GPU version 1.4.0 [24]. Logistic regression required scikit-learn version 0.19.1. We obtained SHAP (SHapley Additive exPlanation) values [25] by executing package ‘shap’ to interpret model predictions. To evaluate the robustness of markers we selected, random sampling of 100 times were carried out. Each time, 6000 samples were random selected from all samples. Each data set was requested to have the same ratio of cancer and normal as that of the original data set. Additionally, <30% sample overlap among all 100 data sets was required too. Each data set was divided into one training data set and one test data set with same ratio of cancer and normal.

## 3. Results

### 3.1. Identifying Cancer-Specific Methylation Markers by Machine Learning

**Figure 1:**
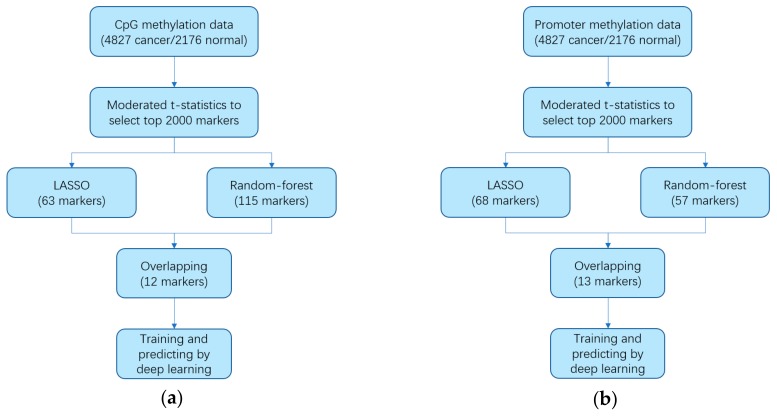
Workflow chart of identifying markers by machine learning. (**a**) Workflow of CpG methylation data. (**b**) Workflow of promoter methylation data. CpG methylation data contained 139,422 CpG sites, and promoter methylation data contained 15,316 promoters. We utilized the Training data set containing 4827 cancer samples and 2176 normal samples to identify markers applying three machine learning strategies (Moderated t-statistics, LASSO, and Random-forest) and obtained 12 markers for the CpG methylation data, and 13 markers for the promoter methylation data. Then, we trained two deep learning models for CpG markers and promoter markers respectively.

We utilized the Training data set to analyze and identify methylation markers by three machine learning methods. Figure 1 shows the procedure of identifying methylation marker. We organized the Training data set into two data matrices: CpG methylation matrix and promoter matrix. The CpG methylation matrix consisted of beta values of 139,422 CpG methylation sites, and promoter matrix consisted of beta values of 15,316 promoters. These two data matrices were utilized to identity the CpG markers and the promoter markers. First, a prescreening procedure was conducted by ‘moderated t-statistics’, to identify candidate markers with the most differential methylation beta value between cancer samples and normal samples. After that, we obtained the top 2000 markers as the candidate markers, including 2000 CpG markers and 2000 promoter markers. Next, we used two strategies to reduce the number of markers parallelly. One machine learning strategy was LASSO (least absolute shrinkage and selection operator) under a binomial distribution. After 1000 times sampling, we selected the markers that were chosen by LASSO at least 750 times. Eventually, by LASSO we got 63 CpG markers and 68 promoter markers. Another machine learning strategy was random forest, and we got 115 CpG markers and 57 promoter markers. We took 12 overlapping CpG markers (Table 1) and 13 overlapping promoter markers (Table 2) between these two machine learning methods as final markers. In 12 CpG markers, reference genes of three markers involve cancer-related pathway. SOX14 (cg04374393 locates at the promoter of SOX14 gene) involves molecular mechanisms of cancer; TP73 (reference gene of cg17804348) involves p53 signaling pathway; SND1 (cg26642667 locates at the promoter of SND1 gene) involves viral carcinogenesis. In 13 promoter markers, associated genes of four markers involve cancer-related pathway. ACVRL1 involves TGF-beta signaling pathway; AURKB involves regulation of TP53 activity; RHOT2 involves mitophagy; WT1 involves transcriptional misregulation in cancer.

### 3.2. Constructing Diagnostic Prediction Models by Deep Learning

**Figure 2:**
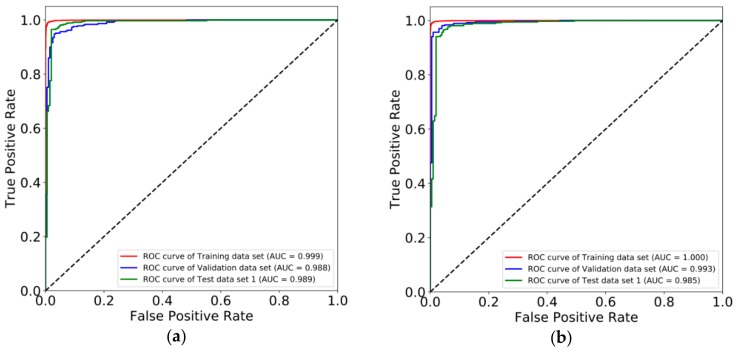
ROC curves of the three data sets. (**a**) ROC curves of CpG methylation data. (**b**) ROC curves of promoter methylation data. ROC curves: Receiver operating characteristic curves.

**Figure 3:**
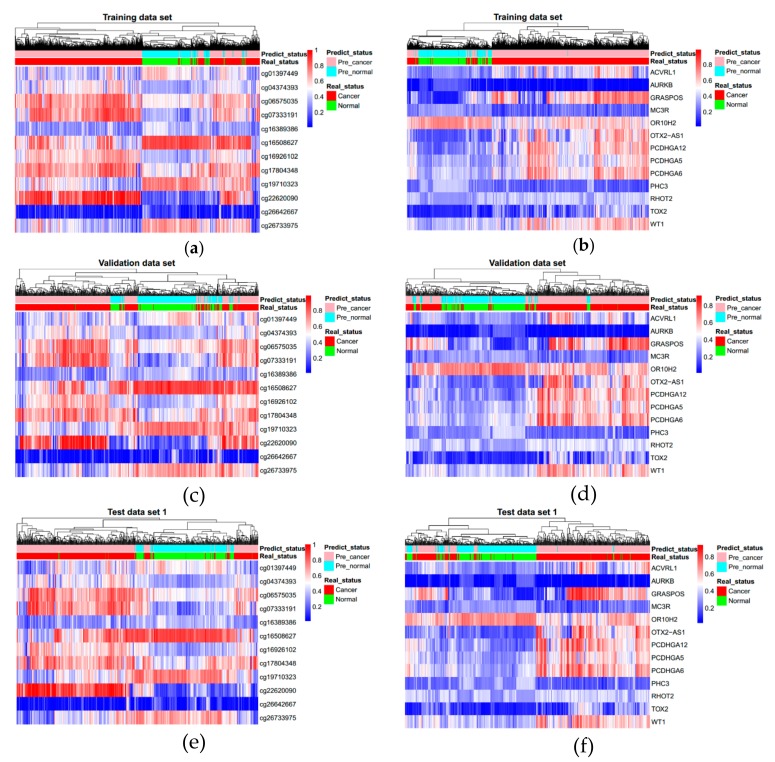
Unsupervised hierarchical clustering of the three data sets. (**a**,**c**,**e**) come from 12 CpG markers and (**b**,**d**,**f**) come from 13 promoter markers. Methylation beta values range from 0 to 1.

**Figure 4:**
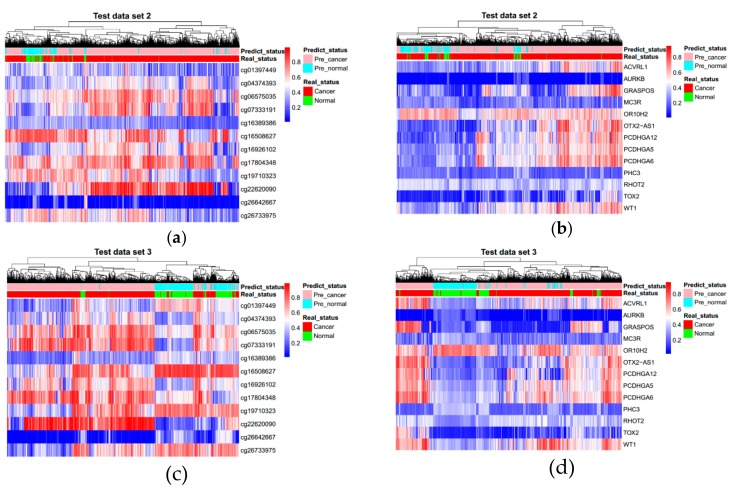
Unsupervised hierarchical clustering of Test data set 2 and Test data set 3. (**a**,**c**) come from 12 CpG markers and (**b**,**d**) come from 13 promoter markers. Methylation beta values range from 0 to 1.

**Figure 5:**
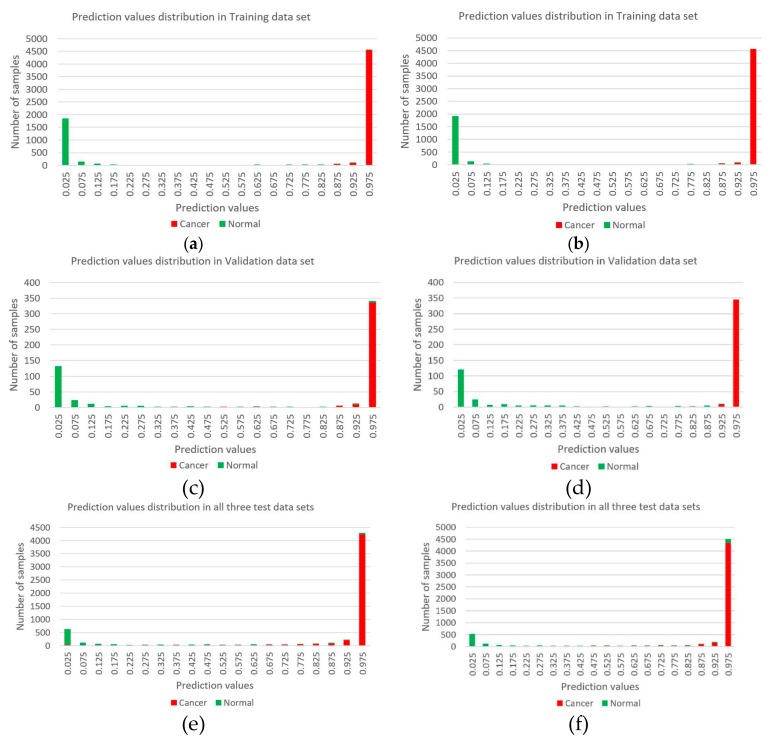
The distribution of prediction values in all samples. (**a**,**c**,**e**) come from 12 CpG markers and (**b**,**d**,**f**) come from 13 promoter markers. Red indicates the status of the sample is cancer, and green indicates the status of the sample is normal.

The markers obtained by machine learning were used to classify and predict cancer and normal samples by deep learning method. We constructed two multi-layer feedforward neural networks based on the deep learning framework Tensorflow and fed the Training data set into these two deep neural network models. We utilized a random search for hyper-parameter optimization, and Table S7 shows the best hyper-parameter combination. These two deep learning models were deployed with the best hyper-parameters and trained again. Figure S1 shows the training curves. By early stopping strategy, we chose a befitting training point to stop, to make the model more suitable for the Validation data set. After obtaining the best parameters, we tested our deep learning models in the three test data sets (Test data set 1, Test data set 2, and Test data set 3). Figure 2 shows the ROC (Receiver operating characteristic) curves of both two marker types. AUC (Area under the Curve of ROC) of Test data set 1 is 0.989 for CpG markers, and 0.985 for promoter markers. Figure 3 shows the results of unsupervised hierarchical clustering for Training data set, Validation data set, and Test data set 1, while Figure 4 shows the results for the other two test data sets (Test data set 2 and Test data set 3). These results indicate that cancer samples can be distinguished markedly from normal samples by both two marker types. Table 3 shows a summary of all prediction results for both CpG markers and promoter markers (More details see Table 4, Table 5, Table 6 and Table 7). Figure 5 shows the distribution of predict values in all samples. For CpG markers, average sensitivity and specificity of three test data sets were 92.8% and 90.1% respectively (Table 3). For promoter markers, average sensitivity and specificity of three test data sets were 89.8% and 81.1% respectively (Table 3). Although sensitivity and specificity in most cancer types were higher than 0.7 for both two marker types, specificity of esophagus and stomach cancer for promoter markers were lower than 0.6, and the sensitivity of oral, thyroid, and nasopharynx cancer for promoter markers were lower than 0.6 (Table 4, Table 5, Table 6 and Table 7). Therefore, all 27 cancer types could be diagnosed precisely by CpG markers, while only twenty-two of 27 cancer types could be diagnosed precisely by promoter markers. Both two categories of markers predicted the same results in each of 88.4% samples (i.e., 5262 samples) of three test data sets (i.e., 5952 samples), and average sensitivity and specificity of these 5262 samples were promoted to 96.4% and 91.6%. Therefore, if the prediction result of one sample is same between CpG markers and promoter markers, the prediction will be more reliable. Average sensitivity and specificity in Test data set 1 were much higher than Test data set 2 and Test data set 3 for both two categories of markers, which means the models we trained are more adapted to eight trained tissue types than the other 20 untrained tissue types. Furthermore, for CpG markers, sensitivity and specificity of the eight cancers (breast, kidney, liver, lung, bile duct, lymph nodes, cervix, and skin cancer) were both higher than 95% (Table 4, Table 5, Table 6 and Table 7). Additionally, for promoter markers, sensitivity and specificity of nine cancers (breast, colorectal, liver, lung, adrenal gland, bile duct, soft tissue, cervix, and skin cancer) were both higher than 95% (Table 4, Table 5, Table 6 and Table 7). Conclusively, the prediction results indicate that our deep learning models can correctly classify cancer samples and normal samples in pan-cancers.

**Figure 6:**
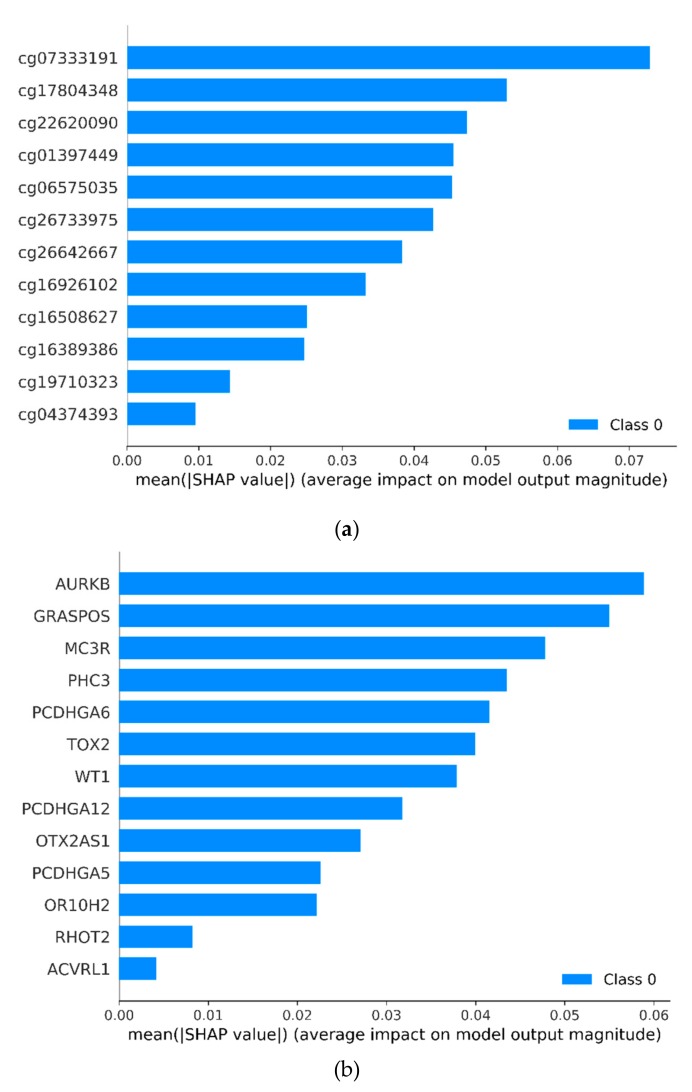
The distribution of average absolute SHAP value for all markers. (**a**) comes from 12 CpG markers and (**b**) comes from 13 promoter markers. SHAP: SHapley Additive explanation.

Interpreting model predictions becomes more and more crucial in the field of machine learning, especially for deep learning. An outstanding approach has been proposed, which used SHAP (SHapley Additive exPlanation) values as a unified measure of feature importance [26]. Figure 6 shows the average absolute SHAP value of each marker. For CpG markers, cg07333191 has biggest impact on model output, while cg04374393 has least impact. For promoter markers, AURKB has biggest impact on model output, while the impact of ACVRL1 is least. Figure S4 shows the detailed impact of each marker to the model output in four samples.

### 3.3. Evaluating Reliability of Markers and Diagnostic Prediction Models

To verify whether our deep learning models perform better than general traditional machine learning strategy, such as logistic regression, we fitted our data in two logistic regression models. The results indicate that deep learning predicting method performs more precise than logistic regression method in our data sets actually (Figure S2, Table S8). To test the reliability of the selected markers, we randomly partitioned all samples into 80% for training, 10% for validation, and 10% for testing. We constructed two other deep learning models for CpG markers and promoter markers, and fed all these samples into the models. Figure S3 shows ROC of the three data sets, and AUCs (0.993 for CpG markers and 0.995 for promoter markers) demonstrate that the selected markers can classify all samples precisely. The robustness of biomarkers for cancer diagnosis or prognosis might be low due to tumor heterogeneity, and random sampling was suggested to evaluate the robustness of markers [27]. We performed random sampling of 100 times, data were divided into one training data set and one test data set each time. Training data set was used to train models and test data set was used to evaluate the performance of models. Figure S5 shows predict accuracies for both CpG markers and promoter markers. The result indicates the robustness of our markers is strong, since the predict accuracies are high in different data sets. To test performance of the markers in liquid biopsy, we utilized the markers to predict cell-free DNA methylation data of 163 prostate cancer samples. Sensitivity for CpG markers was 100%, and for promoter markers was 92%. Additionally, another dataset whose GEO accession number is GSE110185 contains six cell-free DNA pooled samples (two colorectal cancer, two advanced adenomas and two healthy control samples). Additionally, all of these six samples were predicted as normal samples. Notably, for both marker types, specificity of normal whole blood is 100%, and whole blood samples are the most similar samples to cell-free DNA samples.

## 4. Discussion

Most related studies identifying methylation markers focused on one or a few cancer types. The most important impact for our study is that we attempted to identify two categories of methylation markers, CpG markers and promoter markers, to classify and predict pan-cancers. The reliability of this study lies in the fact that all three test data sets were tested only once to avoid overfitting. Therefore, the predict results we show here can prove that pan-cancers can be predicted precisely by the selected methylation markers. Sensitivity and specificity in most cancer types are high enough for both markers. Nonetheless, for promoter markers, specificities of two cancer types (esophagus and stomach cancer) and sensitivities of three cancer types (oral, thyroid, and nasopharynx cancer) are too low to predict precisely. Sensitivity and specificity of these five cancer types are high enough for CpG markers, which means the samples are qualified. Therefore, a possible reason is that in these samples, CpG probes located at promoters are not enough to calculate promoter methylation values precisely. This is the potential defect of promoter markers that promoter methylation value calculating might be inaccurate since each promoter has different length definition actually. Another possible reason is that CpG markers may be more adapted to these cancer types than promoter markers. Nonetheless, identifying promoter methylation markers is worth attempting, since promoter has a close relation with the process of cancer developing. The advantage of pan-cancer methylation biomarkers is that diagnosis of diverse cancer types can be based on targeted measuring of these biomarkers. Therefore, these biomarkers could be applied in liquid biopsy effectively. The performance of the selected markers in cell-free DNA methylation data of 163 cancer samples was excellent. However, for GSE110185 dataset, all six pooled samples were predicted as normal samples. Two advanced adenomas samples should be regarded as non-cancer samples, thus the prediction accuracy is 0.667. However, because lack of abundant normal cell-free DNA samples, specificity remains to be verified in more normal samples. We have put arguments of the well-trained deep learning models online to let more researchers validate the reliability of our model. What should be emphasized is the dependability of cell-free DNA samples. Since in the process of cell-free DNA isolation, contamination could easily happen, such as ruptured blood cells [28]. Therefore, samples containing cell-free DNA are prone to be classified as normal samples. 

Comparing other studies to our study, Vrba et al. [15] attempted to identify CpG markers to predict pan-cancer. One difference between their strategy and ours is that they reduced the number of markers by comparing cancer samples to mix unrelated normal whole blood samples. While we identified markers by comparing cancer samples to mix matched normal samples. Another difference is that they identified markers in each cancer, and summarized all markers to one marker set. However, our strategy involves gathering all samples from the start, and identifying markers fitting all data. Due to a lack of cell-free DNA methylation data, one compromise in their research is that they treated whole blood samples as cell-free DNA samples simulation. Although whole blood samples mainly contained leukocytes, whole blood samples are the most similar samples to cell-free samples. Therefore, specificity for cell-free DNA samples in our study could be calculated by whole blood approximately, which means for both two marker types, specificity of whole blood is 100%. In our study, taking the intersection of markers from two machine learning strategies to reduce the number of markers is a compromising strategy. In the future, more convincing statistics, machine learning, and data dividing strategies for mining marker are necessary. With a lack of abundant cell-free DNA samples, more verification results depend on more researchers using our models published online to classify cell-free DNA samples. Additionally, the pipeline of this study can be applied in cell-free DNA samples to identify methylation markers more adaptive to cell-free DNA samples. The long-range perspective is identifying one methylation markers set for cell-free DNA samples, applying them to cancer early diagnosis for pan-cancers, and making all cancers be exposed early, be cured early, to reduce death rate of cancers. The models we have trained can only diagnose whether a sample is cancer or normal tissue, but cannot judge which cancer type the sample belongs to. Multiple classification models need to be constructed to diagnose the exact cancer type of samples in future study.

## 5. Conclusions

In our study, we collected whole genome methylation data of 10,140 cancer samples and 3386 normal samples, and divided them into five data sets. Using three machine learning methods, we identified two categories of markers: 12 CpG markers and 13 promoter markers. Three of 12 CpG markers and four of 13 promoter markers located at cancer-related genes. The performances of these markers in solid or cell-free DNA samples are both pretty good. Additionally, if the prediction result of one sample is the same between CpG markers and promoter markers, the prediction will be more reliable. To conclude, we found it possible to identify methylation markers used to predict pan-cancer. The long-range perspective is identifying one methylation markers set for efficient and precise liquid biopsy of pan-cancers.

## Figures and Tables

**Table 1:** Characteristics of 12 CpG markers in the Training data set.

Markers	Ref Gene	Coefficients	SE	*z* Value	*p* Value
		4.28017	0.12365	34.614	<0.001
cg01397449	EXOC3L1	−1.26195	0.0828	−15.241	<0.001
cg04374393	SOX14	0.44095	0.10759	4.098	<0.001
cg06575035	PCDHGA1	1.0089	0.09321	10.823	<0.001
cg07333191	Chr4:13	0.5435	0.11389	4.772	<0.001
cg16389386	Chr7:154	−0.38554	0.06408	−6.016	<0.001
cg16508627	HS3ST2	−0.54732	0.11407	−4.798	<0.001
cg16926102	Chr10:23	0.8946	0.11951	7.486	<0.001
cg17804348	TP73	1.09724	0.06442	17.033	<0.001
cg19710323	Chr12:34	−0.8628	0.10259	−8.41	<0.001
cg22620090	Chr6:104	0.36339	0.07759	4.683	<0.001
cg26642667	SND1	−0.85746	0.04911	−17.461	<0.001
cg26733975	RP11–760D2.1	−0.97163	0.10248	−9.481	<0.001

Note: SE indicates standard errors of coefficients; *z* value indicates Wald *z*-statistic value.

**Table 2:** Characteristics of 13 promoter markers in the Training data set.

Markers	Coefficients	SE	*z* Value	*p* Value
	2.6472	0.5316	4.979	<0.001
ACVRL1	5.5848	0.7523	7.423	<0.001
AURKB	−3.9969	1.2242	−3.265	0.001
GRASPOS	−1.0094	0.3599	−2.805	0.005
MC3R	−12.2853	0.858	−14.319	<0.001
OR10H2	−6.8254	0.6101	−11.188	<0.001
OTX2-AS1	3.664	0.6136	5.972	<0.001
PCDHGA12	0.6188	0.5294	1.169	0.242
PCDHGA5	1.8653	0.704	2.649	0.008
PCDHGA6	1.0961	0.6552	1.673	0.094
PHC3	−12.865	0.9678	−13.293	<0.001
RHOT2	11.3143	0.8959	12.628	<0.001
TOX2	3.039	0.8061	3.77	<0.001
WT1	4.5058	0.4796	9.394	<0.001

Note: SE indicates standard errors of coefficients; *z* value indicates Wald *z*-statistic value.

**Table 3:** The summary of all prediction results.

Marker	Data Set	Total	Cancer				Normal				Total Accuracy	MCC
			Cancer Total	Predict Cancer	Predict Normal	Sensitivity	Normal Total	Predict Cancer	Predict Normal	Specificity		
CpG	Training	7003	4827	4734	93	0.981	2176	11	2165	0.995	0.985	0.966
	Validation	571	370	352	18	0.951	201	10	191	0.95	0.951	0.894
	Test set 1	571	370	360	10	0.973	201	9	192	0.955	0.967	0.927
	Test set 2	3309	3041	2795	246	0.919	268	39	229	0.854	0.914	0.602
	Test set 3	2072	1532	1433	99	0.935	540	52	488	0.904	0.927	0.817
	All three test sets	5952	4943	4588	355	0.928	1009	100	909	0.901	0.924	0.761
Promoter	Training	7003	4827	4676	151	0.969	2176	3	2173	0.999	0.978	0.951
	Validation	571	370	354	16	0.957	201	5	196	0.975	0.963	0.921
	Test set 1	571	370	353	17	0.954	201	8	193	0.96	0.956	0.906
	Test set 2	3309	3041	2641	400	0.868	268	28	240	0.9	0.871	0.528
	Test set 3	2072	1532	1443	89	0.942	540	155	385	0.713	0.882	0.684
	All three test sets	5952	4943	4437	506	0.898	1009	191	818	0.811	0.883	0.639

Note: ‘Predict cancer’ or ‘Predict normal’ indicates samples predicted as cancer or normal. Training, Validation, Test set 1, Test set 2 and Test set 3 respectively indicate Training data set, Validation data set, Test data set 1, Test data set 2 and Test data set 3. MCC indicates Matthews Correlation Coefficient [25].

**Table 4:** The prediction results of three data sets for 12 CpG markers.

Data Set	Tissue Types	Total	Cancer				Normal				Total Accuracy	MCC
			Cancer Total	Predict Cancer	Predict Normal	Sensitivity	Normal Total	Predict Cancer	Predict Normal	Specificity		
Training	Breast	1122	1006	993	13	0.987	116	2	114	0.983	0.987	0.932
	Colorectal	390	371	367	4	0.989	19	0	19	1	0.99	0.904
	Kidney	794	593	573	20	0.966	201	0	201	1	0.975	0.937
	Leukocyte	576	0	0	0	-	576	1	575	0.998	0.998	0
	Liver	442	366	355	11	0.97	76	0	76	1	0.975	0.92
	Lung	1155	857	839	18	0.979	298	2	296	0.993	0.983	0.956
	Prostate	529	491	476	15	0.969	38	0	38	1	0.972	0.834
	Uterus	432	416	415	1	0.998	16	0	16	1	0.998	0.969
Validation	Breast	85	60	60	0	1	25	1	24	0.96	0.988	0.972
	Colorectal	56	40	40	0	1	16	2	14	0.875	0.964	0.913
	Kidney	85	60	56	4	0.933	25	0	25	1	0.953	0.897
	Leukocyte	40	0	0	0	-	40	0	40	1	1	0
	Liver	60	40	40	0	1	20	0	20	1	1	1
	Lung	120	80	73	7	0.912	40	1	39	0.975	0.933	0.86
	Prostate	70	50	44	6	0.88	20	5	15	0.75	0.843	0.621
	Uterus	55	40	39	1	0.975	15	1	14	0.933	0.964	0.908
Test 1	Breast	85	60	58	2	0.967	25	1	24	0.96	0.965	0.916
	Colorectal	56	40	40	0	1	16	1	15	0.938	0.982	0.956
	Kidney	85	60	58	2	0.967	25	0	25	1	0.976	0.946
	Leukocyte	40	0	0	0	-	40	0	40	1	1	0
	Liver	60	40	38	2	0.95	20	1	19	0.95	0.95	0.889
	Lung	120	80	80	0	1	40	0	40	1	1	1
	Prostate	70	50	46	4	0.92	20	5	15	0.75	0.871	0.681
	Uterus	55	40	40	0	1	15	1	14	0.933	0.982	0.954

**Table 5:** The prediction results of three data sets for 13 promoter markers.

Data Set	Tissue Types	Total	Cancer				Normal				Total Accuracy	MCC
			Cancer Total	Predict Cancer	Predict Normal	Sensitivity	Normal Total	Predict Cancer	Predict Normal	Specificity		
Training	Breast	1122	1006	984	22	0.978	116	1	115	0.991	0.98	0.902
	Colorectal	390	371	370	1	0.997	19	0	19	1	0.997	0.973
	Kidney	794	593	545	48	0.919	201	0	201	1	0.94	0.861
	Leukocyte	576	0	0	0	-	576	0	576	1	1	0
	Liver	442	366	354	12	0.967	76	0	76	1	0.973	0.914
	Lung	1155	857	829	28	0.967	298	0	298	1	0.976	0.94
	Prostate	529	491	470	21	0.957	38	1	37	0.974	0.958	0.769
	Uterus	432	416	416	0	1	16	1	15	0.938	0.998	0.967
Validation	Breast	85	60	59	1	0.983	25	0	25	1	0.988	0.972
	Colorectal	56	40	40	0	1	16	0	16	1	1	1
	Kidney	85	60	57	3	0.95	25	0	25	1	0.965	0.921
	Leukocyte	40	0	0	0	-	40	0	40	1	1	0
	Liver	60	40	40	0	1	20	0	20	1	1	1
	Lung	120	80	70	10	0.875	40	0	40	1	0.917	0.837
	Prostate	70	50	48	2	0.96	20	3	17	0.85	0.929	0.823
	Uterus	55	40	40	0	1	15	2	13	0.867	0.964	0.909
Test set 1	Breast	85	60	57	3	0.95	25	0	25	1	0.965	0.921
	Colorectal	56	40	40	0	1	16	0	16	1	1	1
	Kidney	85	60	56	4	0.933	25	0	25	1	0.953	0.897
	Leukocyte	40	0	0	0	-	40	0	40	1	1	0
	Liver	60	40	38	2	0.95	20	1	19	0.95	0.95	0.889
	Lung	120	80	77	3	0.963	40	0	40	1	0.975	0.946
	Prostate	70	50	45	5	0.9	20	5	15	0.75	0.857	0.65
	Uterus	55	40	40	0	1	15	2	13	0.867	0.964	0.909

**Table 6:** The prediction results of two test data sets for 12 CpG markers.

Data Set	Tissue Types	Total	Cancer				Normal				Total Accuracy	MCC
			Cancer Total	Predict Cancer	Predict Normal	Sensitivity	Normal Total	Predict Cancer	Predict Normal	Specificity		
Test data set 2	Adrenal gland	267	264	213	51	0.807	3	0	3	1	0.809	0.212
	Bile duct	45	36	36	0	1	9	0	9	1	1	1
	Bladder	440	419	411	8	0.981	21	3	18	0.857	0.975	0.758
	Esophagus	202	186	185	1	0.995	16	5	11	0.688	0.97	0.779
	Eyes	80	80	74	6	0.925	0	0	0	-	0.925	0
	Head and neck	580	530	529	1	0.998	50	10	40	0.8	0.981	0.874
	Lymph nodes	51	48	46	2	0.958	3	0	3	1	0.961	0.758
	Oral	104	65	46	19	0.708	39	2	37	0.949	0.798	0.637
	Ovary	10	10	10	0	1	0	0	0	-	1	0
	Pancreas	391	352	265	87	0.753	39	3	36	0.923	0.77	0.436
	Pleura	87	87	81	6	0.931	0	0	0	-	0.931	0
	Small bowel	56	28	27	1	0.964	28	4	24	0.857	0.911	0.826
	Soft tissue	269	265	250	15	0.943	4	0	4	1	0.944	0.446
	Testis	156	156	135	21	0.865	0	0	0	-	0.865	0
	Thyroid	571	515	487	28	0.946	56	12	44	0.786	0.93	0.655
Test data set 3	Bone marrow	386	325	257	68	0.791	61	0	61	1	0.824	0.611
	Cervix	356	315	311	4	0.987	41	1	40	0.976	0.986	0.934
	Nasopharynx	48	24	19	5	0.792	24	2	22	0.917	0.854	0.714
	Skin	694	473	466	7	0.985	221	1	220	0.995	0.988	0.974
	Stomach	588	395	380	15	0.962	193	48	145	0.751	0.893	0.753

**Table 7:** The prediction results of two test data sets for 13 promoter markers.

Data Set	Tissue Types	Total	Cancer				Normal				Total Accuracy	MCC
			Cancer Total	Predict Cancer	Predict Normal	Sensitivity	Normal Total	Predict Cancer	Predict Normal	Specificity		
Test data set 2	Adrenal gland	267	264	251	13	0.951	3	0	3	1	0.951	0.422
	Bile duct	45	36	36	0	1	9	0	9	1	1	1
	Bladder	440	419	414	5	0.988	21	3	18	0.857	0.982	0.81
	Esophagus	202	186	186	0	1	16	7	9	0.562	0.965	0.736
	Eyes	80	80	74	6	0.925	0	0	0	-	0.925	0
	Head and neck	580	530	523	7	0.987	50	6	44	0.88	0.978	0.859
	Lymph nodes	51	48	48	0	1	3	3	0	0	0.941	0
	Oral	104	65	37	28	0.569	39	2	37	0.949	0.712	0.518
	Ovary	10	10	10	0	1	0	0	0	-	1	0
	Pancreas	391	352	297	55	0.844	39	3	36	0.923	0.852	0.544
	Pleura	87	87	82	5	0.943	0	0	0	-	0.943	0
	Small bowel	56	28	27	1	0.964	28	2	26	0.929	0.946	0.893
	Soft tissue	269	265	263	2	0.992	4	0	4	1	0.993	0.813
	Testis	156	156	134	22	0.859	0	0	0	-	0.859	0
	Thyroid	571	515	259	256	0.503	56	2	54	0.964	0.548	0.279
Test data set 3	Bone marrow	386	325	261	64	0.803	61	0	61	1	0.834	0.626
	Cervix	356	315	310	5	0.984	41	0	41	1	0.986	0.937
	Nasopharynx	48	24	9	15	0.375	24	0	24	1	0.688	0.48
	Skin	694	473	470	3	0.994	221	1	220	0.995	0.994	0.987
	Stomach	588	395	393	2	0.995	193	154	39	0.202	0.735	0.363

## Supplementary Materials

The following are available online at https://www.mdpi.com/2073-4425/10/10/778/s1, Figure S1: The cost and accuracy change in training progress, Figure S2: ROC of logistic regression for the three data sets, Figure S3: ROC of the randomly partition three data set, Figure S4: The detailed impact of each marker to the model output in four samples, Figure S5: Plot of accuracy with random sampling, Table S1: The overview of Training data set, Validation data set, and Test data set 1, Table S2: The sample details of 4840 cancer samples and 1742 matched normal samples (All were divided into Training data set, Validation data set and Test data set 1 as Table S1 shows), Table S3: The sample details of 727 cancer samples and 836 normal samples (All were added into Training data set), Table S4: The sample details of 3041 cancer samples and 268 matched normal samples (All were added into Test data set 2), Table S5: The sample details of 1532 cancer samples and 540 virtual normal samples (All were added into Test data set 3), Table S6: The sample details of cell-free DNA samples, Table S7: The range of hyper-parameters during random searching, Table S8: The prediction accuracy contrast between Deep learning and Logistic regression.
