# Supplementary material for: DNA Methylation Markers for Pan-Cancer Prediction by Deep Learning
Source: Genes (Basel). 2019 Oct 4;10(10):778. doi: 10.3390/genes10100778 (PMC6826785; doi:10.3390/genes10100778)
Supplement: Supplementary file 1 [file genes-10-00778-s001.zip › genes-548261-supplementary/genes-548261-supplementary.dot]

**Table S1.** The overview of Training data set, Validation data set, and Test data set 1.

| **Tissue types** | **Cancer** | | | | **Normal** | | | | **Total** |
| --- | --- | --- | --- | --- | --- | --- | --- | --- | --- |
|  | **Cancer total** | **Training** | **Validation** | **Test** | **Normal total** | **Training** | **Validation** | **Test** |  |
| Breast | 1126 | 1006 | 60 | 60 | 166 | 116 | 25 | 25 | 1292 |
| Colorectal | 451 | 371 | 40 | 40 | 51 | 19 | 16 | 16 | 502 |
| Kidney | 713 | 593 | 60 | 60 | 251 | 201 | 25 | 25 | 964 |
| Leukocyte | 0 | 0 | 0 | 0 | 656 | 576 | 40 | 40 | 656 |
| Liver | 446 | 366 | 40 | 40 | 116 | 76 | 20 | 20 | 562 |
| Lung | 1017 | 857 | 80 | 80 | 378 | 298 | 40 | 40 | 1395 |
| Prostate | 591 | 491 | 50 | 50 | 78 | 38 | 20 | 20 | 669 |
| Uterus | 496 | 416 | 40 | 40 | 46 | 16 | 15 | 15 | 542 |
| Total | 4840 | 4100 | 370 | 370 | 1742 | 1340 | 201 | 201 | 6582 |

Note: Training, Validation and Test indicate Training data set, Validation data set, and Test data set 1 respectively.

**Table S7.** The range of hyper-parameters during random searching.

| **Hyper-parameters** | **Range** | **Best scheme for CpG markers** | **Best scheme for promoter markers** |
| --- | --- | --- | --- |
| Layers | 1;2;3;4;5;6;7 | 3 | 3 |
| Units number | 20-120 | 100 | 100 |
| Minibatch size | 32;64;128;256;512;1024 | 256 | 256 |
| Learning rate base | 0.00001-1 | 0.00001 | 0.00001 |
| Regularization rate | 0.0001-1 | 0.1 | 0.1 |
| Decay steps | 200;400;600;800;1000 | 600 | 800 |
| Decay rate | 0.5-0.9 | 0.9 | 0.8 |

|   (**a**) |   (**b**) |
| --- | --- |
|   (c) |   (d) |

**Figure S1.** The cost and accuracy change in training progress. (a, c) come from 12 CpG markers, and (b, d) come from 13 promoter markers. The cost values are namely the calculating values of cost function in deep learning models. The accuracy values equal number of samples predicted correctly dividing the number of total samples.

|   (**a**) |   (**b**) |
| --- | --- |

**Figure S2.** ROC of logistic regression for the three data sets. (a) comes from 12 CpG markers, and (b) comes from 13 promoter markers.

|   (**a**) |   (**b**) |
| --- | --- |

**Figure S3.** ROC of the randomly partition three data set. (a) comes from 12 CpG markers, and (b) comes from 13 promoter markers.

(**a**)

(**b**)

(**c**)

(d)

**Figure S4.** The detailed impact of each marker to the model output in four samples. (a, b) come from 12 CpG markers, and (c, d) come from 13 promoter markers. (a) The model output value is 1 and the sample is predicted as cancer. The biggest impact to output value comes from cg07333191. (b) The model output value is 0 and the sample is predicted as normal. The biggest impact to output value comes from cg07333191. (c) The model output value is 1 and the sample is predicted as cancer. The biggest impact to output value comes from PCDHGA6. (d) The model output value is 0 and the sample is predicted as normal. The biggest impact to output value comes from AURKB.

**Figure S5.** Plot of accuracy with random sampling. For both CpG markers and promoter markers, random sampling of 100 times were performed. Data were divided into training data set and test data set each sampling to evaluate the robustness of markers.

**Table S8.** The prediction accuracy contrast between Deep learning and Logistic regression.

| **Data set** | **CpG markers** | | **Promoter markers** | |
| --- | --- | --- | --- | --- |
|  | **Deep learning** | **Logistic regression** | **Deep learning** | **Logistic regression** |
| Training data set | 0.985 | 0.971 | 0.978 | 0.972 |
| Validation data set | 0.951 | 0.926 | 0.963 | 0.918 |
| Test data set 1 | 0.967 | 0.944 | 0.956 | 0.918 |
| Test data set 2 | 0.914 | 0.878 | 0.871 | 0.79 |
| Test data set 3 | 0.927 | 0.91 | 0.882 | 0.88 |
| All three test data sets | 0.924 | 0.895 | 0.883 | 0.833 |
